# Evaluation of Glutathione S-Transferase *GSTM1* and *GSTT1* Deletion Polymorphisms on Type-2 Diabetes Mellitus Risk

**DOI:** 10.1371/journal.pone.0076262

**Published:** 2013-10-03

**Authors:** Denise S. Pinheiro, César R. Rocha Filho, Cláudia A. Mundim, Paulo de Marco Júnior, Cirano J. Ulhoa, Angela A. S. Reis, Paulo C. Ghedini

**Affiliations:** 1 Institute of Biological Sciences (ICB), Federal University of Goiás (UFG), Goiânia-GO, Brazil; 2 Institute of Tropical Pathology (IPTSP), Federal University of Goiás (UFG), Goiânia-GO, Brazil; 3 Endocrinology Service, Clinic Hospital (HC), Federal University of Goiás (UFG), Goiânia-GO, Brazil; 4 Department of Biochemistry and Molecular Biology (DBBM), Institute of Biological Sciences (ICB), Federal University of Goiás (UFG), Goiânia-GO, Brazil; 5 Department of Ecology (DE), Institute of Biological Sciences (ICB), Federal University of Goiás (UFG), Goiânia-GO, Brazil; 6 Department of Physiological Sciences (DCIF), Institute of Biological Sciences (ICB), Federal University of Goiás (UFG), Goiânia-GO, Brazil; Medical University Innsbruck, Austria

## Abstract

**Background:**

Due to the activity of GSTs in the detoxification of oxidative stress products, deletion polymorphisms of *GSTM1* and *GSTT1* may contribute to susceptibility to T2DM, since B-cells express very low levels of antioxidant enzymes. Recently, some studies have shown an association between *GSTM1*-null/*GSTT1*-null genotypes and an increased susceptibility to T2DM. A relationship between these polymorphisms and changes in the clinical parameters of diabetic patients has also been investigated. However, the results diverge considerably among the studies. Thus, this case-control study was designed to contribute to existing knowledge, as there are no studies on this issue performed in the Brazilian population.

**Methods and Findings:**

A total of 120 patients and 147 healthy individuals were included in this study. *GSTT1* and *GSTM1* deletion polymorphisms were genotyped by multiplex SYBR Green Real-Time PCR. The *GSTT1*-null genotype conferred a 3.2-fold increased risk to T2DM relative to the present genotype. There was no association between *GSTM1*-null and T2DM risk. In diabetic patients, *GSTT1*-null conferred higher levels of triglycerides and VLDL-cholesterol, while *GSTM1*-null was associated with increased levels of fasting blood glucose, glycated hemoglobin and blood pressure. We emphasized a necessity for applying log-linear analysis in order to explore an interaction between these polymorphisms properly.

**Conclusion:**

These results suggest that the *GSTT1* polymorphism may play an important role in the pathogenesis of T2DM in the Brazilian population. This gene could then be added to a set of genetic markers to identify individuals with an increased risk for developing T2DM and complications associated with dyslipidemia in diabetic patients. Although there was no association of *GSTM1* deletion polymorphism with susceptibility to T2DM, the influence of this polymorphism on important clinical parameters related to glycemia and blood pressure levels was verified. This finding suggests that both *GSTM1*-null and *GSTT1*-null may contribute to the clinical course of T2DM patients.

## Introduction

Diabetes Mellitus Type 2 (T2DM) is a multifactorial disease that develops through an exposure to environmental risk factors, lifestyle habits and genetic susceptibility. This heterogeneous syndrome is characterized by chronic hyperglycemia and other metabolic alterations. These mainly include dyslipidemia and hypertension, that leads to a development of macro and microvascular complications. The disease pathogenesis involves a combination of β-cell insufficiency and insulin resistance. Much like other multifactorial diseases, little is known about the genetics of T2DM [1]–[2].

Oxidative stress, as a potential cause of cellular dysfunction, may be related to the pathophysiology of various diseases including cancer, atherosclerosis and diabetes mellitus [3]. Pancreatic β cells are more sensitive to cytotoxic stress than several other cells due to an expression of very low levels of antioxidant enzymes [4]–[6]. It is well documented that oxidative stress is increased in diabetic conditions and is probably involved in pancreatic B-cell dysfunction found in T2DM along with its related complications [5]–[8].

The GSTs are a multigenic superfamily of detoxification enzymes that are essential for cell protection against oxidative damage, as well as the biotransformation of xenobiotics, due to their acting on a wide variety of substrates, mediating the conjugation of reduced glutathione to electrophilic species which leads to the elimination of toxic compounds [9]–[10].

Among the primary human classes of this system, *GSTM1* and *GSTT1* genes exhibit a deletion polymorphism that leads to a lack of active isoforms when in homozygosis, known as the null genotype [9]. In the case of *GSTT1*-null, which occurs at frequencies of 11–38% in different populations, 50 kb of genomic sequence containing the entire gene is deleted. While for the *GSTM1*-null, variable frequencies have a range of 20–70%, involving a 15-kb sequence deletion [8]–[11].

According to the reviewed literature, few studies have been published on the association between *GSTT1*/*GSTM1* polymorphism and susceptibility to diabetes, and there are large divergences among the study results. These range from significant associations by only one of the polymorphisms to diabetes, by both of them, or by neither [12]. This first case-control study on the Brazilian population was designed to provide more information about the effects of the *GSTT1* and *GSTM1* polymorphisms on T2DM risk and the complications associated with this disease. We have obtained important results regarding the contribution of GST polymorphisms to T2DM.

## Patients and Methods

### Ethics Statement

This study was approved by the Ethics in Research Committee at the Federal University of Goiás under protocol number 195/11 dated 27^th^ June, 2011 and was conducted according to the Ethical Principles for Medical Research Involving Human Subjects of the World Medical Association Declaration of Helsinki.

### Subjects

We included a case group of 120 patients with a diagnosis of T2DM being monitored by the Clinical Hospital (Goiânia-GO, Brazil), with a minimum follow-up period of two years. The control group included 147 healthy individuals selected from the general population of our region. All individuals selected for this study were classified into non-white and non-black from the Central Brazil region. In order to obtain homogeneity with respect to gender proportion and the range of ages, we selected approximately two women for every man in a range of ages above 30 years old. Written informed consent was obtained from each participant after a brief and clarified explanation about the survey. Data on lifetime, occupational history, smoking history, general health conditions, previous diseases and other anamnesis data were obtained through interviews.

### Genotyping of *GSTM1* and *GSTT1* Polymorphisms

Peripheral blood samples were collected in tubes containing heparin and stored at −20°C. DNA was extracted by using the Illustra Blood Genomic Prep Mini Spin Kit (GE Healthcare®, USA). For the determination of the polymorphism of *GSTM1* and *GSTT1*, the null alleles were analyzed using a multiplex Real-Time PCR (SYBR Green) and melting curve analysis, as previously described [13]. The final Real-Time PCR protocol was adjusted for use in the IQ5 PCR Thermal Cycler instrument (BioRad®, Minnesota, USA) and used the *RH92600* gene as a reference gene (primers as previously reported) [14]. This molecular method is only able to detect the present genotype (at least one allele present, homozygote or heterozygote) and the null genotype (complete deletion of both alleles, homozygote). Moreover, results obtained by the Real-Time PCR were confirmed by the conventional multiplex PCR and gel electrophoresis, as the protocol anteriorly described [14].

### Statistical Analysis

Statistical analyses were computed using Biostat version 5.3 software. The demographic and clinical characteristics of the participants (T2DM and control groups) were compared using a chi-square test and Fisher's exact test was applied when necessary. The odds ratios (OR) with their corresponding 95% confidence intervals (CI) and p-values were calculated in order to estimate the risk of having T2DM by multiple logistic regression with control for confounding factors related to distribution by sex, smoking and alcohol consumption. The t test was used to compare clinical variables between the groups. Multi-way log-linear analyses were performed to verify the effect of combined genotypes of *GSTM1* and *GSTT1* determined by chi-square test statistics generated by successive hierarchical models. All tests were conducted at the p<0.05 level of significance.

## Results

We included 120 patients with T2DM (83 female and 37 male, 58.98±9.79 years old) and 147 diabetes-free subjects (96 female and 51 male, 60.48±9.88 years old) in this study. The mean age was 60.5 years for patients and 59 years for the control group. Demographic and clinical characteristics of case subjects and controls are shown in Table 1. All clinical variables of the patient group presented values that are significantly altered compared to the controls.

**Table 1:** Characteristics of the study population and a comparison of case and control groups.

Variables	Controls	Cases	P value
Age (years)	58.98±9.79	60.48±9.88	0.2261
Gender (F/M)	96/51	83/37	0.5915
Fasting Blood Glucose (mmol/l)	4.85±0.49	10.32±4.33	<0.0001*
Total Cholesterol (mmol/l)	4.02±0.77	4.76±1.14	<0.0001*
Triglycerides (mmol/l)	1.40±0.47	1.77±1.03	0.0004*
HDL (mmol/l)	1.30±0.26	1.20±0.33	0.0081*
LDL (mmol/l)	2.08±0.73	2.85±0.90	<0.0001*
VLDL (mmol/l)	0.64±0.21	0.80±0.47	0,0004*
BMI (kg/m^2^)	27.35±5.65	28,33±5.57	0.0358*
Diastolic Blood Pressure (kPa)	10.08±1.06	11.08±1.04	<0.0001*
Systolic Blood Pressure (kPa)	16.38±1.42	18.17±1.39	<0.0001*
Smoking Habit [N (%)]	57 (38.78)	57 (47.5)	0.1904
Alcohol Consumption [N (%)]	43 (24.3)	29 (24.2)	0.4279

Data are reported as mean ± standard deviation. Statistical analysis by t test or chi-square.

* Significant difference between groups (p<0.05).

Statistical analysis of the distribution by sex and age showed no significant differences, indicating that there is homogeneity between groups. The proportion of smokers and drinkers showed no significant differences between the two groups. Only those who smoked for at least a year of their life before diagnosis of T2DM in the case group were considered smokers. As for drinkers, all subjects participating in the study reported drinking only occasionally or socially.

Table 2 shows the distribution of the *GSTM1* and *GSTT1* genotypes in T2DM patients and controls. A total of 267 subjects (120 patients and 147 controls) were genotyped for the deletion polymorphism of two GST isoforms. In diabetic patients, the frequency of *GSTT1-*null and *GSTM1*-null were 29.2% and 41.7%, respectively, whereas for the control group, the frequencies of *GSTM1-*null and *GSTT1*-null were 12.2 and 43.5%, respectively (Table 2).

**Table 2:** Distribution of genotypic frequencies for *GSTM1* and *GSTT1* in the study population and a risk analysis of T2DM.

	Case	Control				
Genotype	n (%)	n (%)	χ^2^	P value	OR (95%CI)	P value
***GSTM1***						
Present (+)	70 (58.3)	83 (56.5)	–	–	1 (Reference)	–
Null (−)	50 (41.,7)	64 (43.5)	0.095	0.855	1.1 (0.66–1.82)	0.732
***GSTT1***						
Present (+)	85 (70.8)	129 (87.8)	–	–	1 (Reference)	–
Null (–)	35 (29.2)	18 (12.2)	11.89	0.001*	3.2 (1.68–6.18)	0.0004*
**Total**	120 (100.0)	147 (100.0)				

Analysis by chi-square and multiple logistic regression to obtain adjusted-oddsratio values (OR) and confidence intervals (95% CI).

* Significant difference between groups (p<0.05).

For an analysis of the risk associated with the deletion polymorphism for *GSTT1*, it was found that the null genotype (p = 0.0004) is related to an increased predisposition for T2DM (Table 2), conferring a 3.2-fold increased risk of developing the disease relative to the present genotype. It was also observed that there wasn’t any association of the *GSTM1* deletion with susceptibility to disease (p = 0.732) in the population studied (Table 2).

In Table 3, the distribution analysis for the both *GSTT1* and *GSTM1* genotypes can be observed. There was a low frequency of individuals who had a double null genotype (−/−), for both case and control groups (6.7 and 4.1%, respectively). A higher prevalence of individuals with a double present genotype (+/+) for both groups (35.8 and 48.3%, respectively) was also observed. It was possible to verify a significant difference only for the genotype combination *GSTT1*-null and *GSTM1-*present (−/+), which had a higher proportion in the case group (p = 0.0018). This was associated with an increased 4.14-fold risk to T2DM (p = 0.0005) compared to the double present genotype (+/+).

**Table 3:** Distribution frequencies of genotype combinations between *GSTM1* and *GSTT1* in case and control groups and a risk analysis of T2DM.

Genotype	Case	Control				
*GSTT1/GSTM1*	n (%)	n (%)	χ^2^	P value	OR (95%CI)	P value
(+/+)	43 (35.8)	71 (48.3)	3.70	0.054	1 (Reference)	–
(−/+)	27 (22.5)	12 (8.2)	9.77	0.002*	4.14 (1.85–9.27)	0.0005*
(+/−)	42 (35.0)	58 (39.4)	0.39	0.535	1.22 (0.70–2.14)	0.477
(−/−)	8 (6.7)	6 (4.1)	0.44	0.505	2.51 (0.82–7.75)	0.108
**Total**	120 (100.0)	147 (100.0)				

Analysis by chi-square and multiple logistic regression to obtain adjusted odds ratio values (OR) and confidence intervals (95% CI).

* Significant difference between groups (p<0.05).

The application of loglinear analysis for these data allowed us to infer that there was not an interaction between the two polymorphisms for susceptibility to T2DM (χ^2^ = 1.04, DF = 1, p = 0.309) or an isolated effect of *GSTM1* (χ^2^ = 1.097, DF = 2, p = 0.578). Otherwise, there was a significant effect of *GSTT1* on T2DM (χ^2^ = 12.68, DF = 2, p = 0.002). These results allow us to conclude that *GSTM1* did not contribute to disease susceptibility, and, therefore, the significant odds ratio value obtained in the genotype combination *GSTT1*-null and *GSTM1*present was due only to the effect of *GSTT1*-null on the disease risk.

The influence of *GSTT1* and *GSTM1* deletion on clinical and biochemical alterations was analyzed in the group of patients that were studied by comparing individuals with null and present genotypes. It was found that the risk genotype (null) of *GSTT1* is associated with significantly higher levels of triglycerides (p = 0.024) and VLDL cholesterol (p = 0.025) relative to the present genotype in individuals with T2DM (Table 4).

**Table 4:** Association between null and present genotypes of *GSTT1* with clinical variables in diabetic patients.

*GSTT1*	Present	Null	P value
Gender (M/F)	27/58	10/25	0.899
Fasting Blood Glucose (mmol/l)	10.19±4.17	10.66±4.77	0.590
HbA_1c_ (proportion)	0.09±0.03	0.09±0.03	0.348
BMI (kg/m^2^)	28.96±5.45	28.43±5.82	0.635
Total Cholesterol (mmol/l)	4.68±1.04	4.96±1.35	0.229
Triglycerides (mmol/l)	1.64±0.93	2.10±1.19	0.024*
HDL (mmol/l)	1.20±0.33	1.19±0.34	0.871
LDL (mmol/l)	2.78±0.83	3.02±1.05	0.176
VLDL (mg/dl)	0.74±0.43	0.95±0.54	0.025*
Diastolic Blood Pressure (kPa)	11.12±1.44	10.99±1.51	0.668
Systolic Blood Pressure (kPa)	18.26±2.55	17.97±2.44	0.564
Smoking Habit (+/−)	43/42	14/21	0.393
Alcohol Consumption (+/−)	18/67	11/24	0.237

Data are reported as mean ± standard deviation. Statistical analysis by t test or chi-square.

* Significant difference between groups (p<0.05).

With respect to the *GSTM1* polymorphism, it was observed that despite the fact that it did not have T2DM susceptibility, this isoform null genotype was associated with significantly increased levels of fasting blood glucose (p = 0.048), glycated hemoglobin (0.023) and blood pressure, both systolic (p<0.0001) and diastolic (p<0.0001) when compared to the present genotype in diabetic individuals (Table 5).

**Table 5:** Association between null and present genotypes of *GSTM1* with clinical variables in diabetic patients.

*GSTM1*	Present	Null	P value
Gender (M/F)	19/51	18/32	0.404
Fasting Blood Glucose (mmol/l)	9.66±4.47	11.25±3.99	0.048*
HbA_1c_ (%)	0.09±0.03	0.10±0.03	0.023*
BMI (kg/m^2^)	28,6±4,96	29,1±6,31	0.622
Total Cholesterol (mmol/l)	4.66±1.13	4.91±1.15	0.232
Triglycerides (mmol/l)	1.72±1.00	1.85±1.07	0.497
HDL (mmol/l)	1.21±0.36	1.18±0.30	0.629
LDL (mmol/l)	2.77±0.83	2,97±0.99	0.229
VLDL (mmol/l)	0.79±0.46	0.84±0.49	0.492
Diastolic Blood Pressure (kPa)	10.50±1.18	11.90±1.42	<0.0001*
Systolic Blood Pressure (kPa)	17.23±1.99	19.49±2.58	<0.0001*
Smoking Habit (+/−)	33/37	24/26	0.926
Alcohol Consumption (+/−)	17/53	12/38	0.972

Data are reported as mean ± standard deviation. Statistical analysis by t test or chi-square.

* Significant difference between groups (p<0.05).

## Discussion

In this molecular epidemiologic case-controlled study, *GSTM1* and *GSTT1* deletion polymorphisms were evaluated for their association with susceptibility to T2DM and any complications that may have accompanied the disease by the analysis of clinical data in patients and controls. We demonstrated an association of the *GSTT1*-null genotype with an increased risk of T2DM. This finding was confirmed after adjustment for sex, smoking and alcohol use. Furthermore, there was not a significant association of these confounding factors with a T2DM risk.

In a comparison between the case and control groups, the findings for significant differences in all clinical variables that were analyzed can be explained by a high degree of metabolic decompensation within patients that were involved in the study, as can be seen in Table 1 for the very altered values of the case group variables, especially fasting glucose, total cholesterol, LDL-cholesterol and blood pressure. These findings also demonstrate the complex nature of T2DM. This is recognized as a syndrome that involves alterations in various clinical parameters, which need to be constantly monitored in diabetic patients in order to avoid the occurrence of micro- and macro-vascular complications.

The proportion of subjects with the *GSTM1*-null genotype in the control group (43.5%) is in agreement with the literature data, where similar frequencies of this proportion were reported in case-controlled studies in Brazil [15]–[16] (45.7% and 41.3%, respectively) and in Europe [17] (46.9%). The deletion frequency of *GSTT1* in the control group (12.2%) was relatively lower than the frequencies obtained in these studies that varied from 18 to 20%, which may be due to ethnic differences among regions of the Brazilian population [11] and differences in the exclusion criteria of one control group that focused on cancer.

Additionally, diabetic patients showed a higher frequency of the *GSTT1*-null genotype (29.2%) than healthy subjects (12.2%). Our study showed that the *GSTT1-*null genotype resulted in a statistically significant 3.2-fold increased risk for T2DM (p = 0.0004). Thus, individuals may have decreased antioxidant defenses when this isoform was deleted. Furthermore, it has been well documented that a *GSTT1-*present genotype can confer protection against the development of a T2DM [10–18–19]. These results suggest that the *GSTT1* deletion polymorphism may play a role in the pathogenesis of T2DM. On the order hand, *GSTM1* polymorphism showed no significant differences in genotypic proportions between case (null genotype of 41.7%) and control groups (43.5% of null genotype). It was also found that there was no association of *GSTM1* with susceptibility to T2DM.

There are studies that reported significant association to T2DM for both null genotypes of GST [19], [20] and others that verified no association between GSTT1 and GSTM1 polymorphisms and T2DM [18], [21]. In addition, others studies showed that only the GSTM1-null genotype may play a significant role in the etiopathogenesis of T2DM [22], [23]. In the Turkish population study [22], the authors suggested that the GSTM1 gene may be a useful marker in the prediction of T2DM susceptibility. The OR obtained for the GSTM1-null genotype was 3.7, indicating an association between the incidence of diabetes and GSTM1 deletion polymorphism. In accordance, an Indian population study reported a significant association of GSTM1-null (OR = 2.042) with T2DM and no significant association with GSTT1 [23].

Despite some divergence in the literature data, *GSTT1*-null and *GSTT1*-null/*GSTM1-*null genotypes have consistently been considered risk factors for the development of T2DM as reported by a meta-analysis study [12]. In an Egyptian study [19], the authors found significant differences between the double present genotype (+/+) and either or both null genotypes of diabetics (P = 0.002 and P = 0.009 respectively) when compared to the control subjects. They affirm these results support the notion that *GSTT1* and *GSTM1* cooperatively play a protective role against the development of T2DM. Furthermore, in the Indian study [20], the results implied that there was a 1.84 increased risk for T2DM with the combination of either null genotypes of *GSTM1*/*GSTT1* (+/− or −/+).

We found that subjects with a combined *GSTT1*-null/*GSTM1*-present (−/+) genotype had a statistically significantly 4.14 fold increased susceptibility to disease (p = 0.0005) compared to the double present genotype (+/+) (Table 3). However, the log-linear analysis revealed that there isn’t a contribution of *GSTM1* polymorphism in susceptibility to diabetes (p = 0.578), and moreover, we can conclude that *GSTM1*- present may not buffer a deficiency caused by *GSTT1*-deletion, since the risk to T2DM remained high.

The log-linear analysis allows for checking interactions between categorical variables in a more complete and easier way than the application of a chi-square test [24]–[25], and it should be highlighted that it was noted in the surveyed literature that this tool is rarely used. Findings achieved in this study have demonstrated the need for using a log-linear analysis in susceptibility studies to verify the actual significance of the combined genetic polymorphisms that were analyzed.

The evaluation of clinical variables association with GST polymorphism in diabetic patients showed that the *GSTT1*-null genotype relates to significantly higher levels of triglycerides and VLDL-cholesterol, whereas the *GSTM1*-null genotype correlates with a significant increase in glycated hemoglobin, fasting glucose and systolic and diastolic blood pressure levels when compared to the present genotype. This allows us to infer that the absence of *GSTM1* and *GSTT1* may contribute to type 2 diabetes-related complications, such as dyslipidemia (*GSTT1*), glycemic de-compensation and hypertension (*GSTM1*). These results are consistent with studies conducted on the Chinese population [10], Egyptian population [19] and Indian population [20], where a *GSTT1*-null association with lipid alterations was also observed, and the *GSTM1* influence on glycemic and pressure levels is in agreement with a study conducted on the Egyptian population [19].

The theory that considers hyperlipidemia as a primary cause of B-cell dysfunction instead of hyperglycemia [26] (as it is reported that elevated free fatty acids and other lipids can impair B-cell function) is corroborated by the findings that GSTM1 does not influence the risk of T2DM but instead alters glycemic control along with GSTT1 polymorphism association with increased susceptibility to T2DM with only an alteration to lipid metabolism. Thus, the *GSTT1* gene could be added to a set of potential genetic markers to identify individuals at increased risk for developing T2DM and complications associated with dyslipidemia in diabetic patients.

While a relationship between *GSTM1* deletion polymorphism and susceptibility to disease was not verified, it was possible to observe the influence of this polymorphism on clinical parameters related to blood pressure and blood glucose. Therefore, the deletion of *GSTM1*, as well as *GSTT1*, can have relevance in the clinical course of diabetic patients, since those two variables, along with lipid profile, are focal points for disease monitoring to prevent T2DM complications. The mechanisms underlying the results of association obtained in this and other works still need to be investigated with further research.
